# Clinical utility of skin perfusion pressure measurement in diabetic foot wounds: An observational study

**DOI:** 10.1097/MD.0000000000030454

**Published:** 2022-09-09

**Authors:** Hak Jun Kim, Woo Jong Kim, Hong Seop Lee, Yeong Yoon Koh, Young Bin Shin, Eui Dong Yeo

**Affiliations:** a Department of Orthopaedic Surgery, Korea University Guro Hospital, Seoul, Republic of Korea; b Department of Orthopaedic Surgery, Soonchunhyang University, Cheonan Hospital, CheonanRepublic of Korea; c Department of Orthopaedic Surgery, Nowon Eulji Medical Center, Eulji University, Seoul, Republic of Korea; d Department of Orthopedic Surgery, VHS Medical Center, Seoul, Republic of Korea.

**Keywords:** CT angiography, diabetic foot, skin perfusion pressure, skin ulcer

## Abstract

The degree of blood vessel stenosis significantly influences diabetic foot treatment. This study aimed to investigate the association between computed tomography angiography (CTA) stenosis and skin perfusion pressure (SPP), which are noninvasive vascular assessments used to evaluate diabetic foot wounds.

Forty patients who reported diabetic foot wounds between November 2016 and December 2017 were included in the study. SPPand CTA were performed to evaluate the blood flow, and the rate of decrease in wound size was measured for the wounds corresponding to Meggitt–Wagner grade 1 at the first evaluation and 4-week intervals.

The *P* value of the association between the degree of CTA stenosis and the SPP value was 0.915, and the *P* value of the association between CTA stenosis and decreasing rate of wound size was .235. There was no statistically significant association between SPP and the decreasing rate of wound size according to the degree of CTA stenosis. The association between SPP value and the decreasing rate of wound size was statistically significant (*P* < .05).

The decreasing rate in diabetic foot wound size was significantly associated with SPP but not with CTA stenosis.

## 1. Introduction

Diabetic foot wounds affect 15% to 25% of diabetes patients during their lifetime, and 50% to 70% of wounds recur within 5 years.^[1]^ Diabetic foot wounds are mainly caused by diabetic neuropathy and peripheral vascular diseases.^[1]^ Meggitt–Wagner classification is most commonly used to evaluate diabetic foot wounds, which grades the wounds from 0 to 5 depending on the depth and the degree of necrosis. There are many classifications to evaluate diabetic foot wounds, but none are absolute.^[2]^

Treatment of wounds in diabetic feet is challenging. More than 50% of patients with diabetic foot wounds have peripheral vascular diseases.^[3]^ Specifically, assessing the degree of arterial stenosis, which is known to impact wound healing significantly, is vital for evaluating treatment outcomes and prognosis.^[4]^ Computed tomography angiography (CTA) is a historically and commonly used method to evaluate the vessel condition because it is noninvasive, with a sensitivity and specificity of 91% and 99%, respectively, for peripheral vascular stenosis.^[5]^ However, because CTA requires a contrast medium, its use is limited by the risk of renal dysfunction or allergy to the contrast medium. It is difficult to determine the degree of stenosis of the small arteries.^[6]^ Skin perfusion pressure (SPP) can help assess the severity of ischemia. SPP was introduced in 1967 and is measured using 3 methods: radioisotope clearance, photoplethysmography, and laser Doppler.^[7]^ In SPP, when the blood vessel is reperfused after occlusion, the laser Doppler sensor detects the red blood cells. The SPP methods are not widely used in many practical situations.^[8]^ Moreover, there is a lack of concrete data supporting the SPP method, and there remains a debate over which value should be set as the reference value. This study used laser Doppler imaging for SPP measurement.

We aimed to investigate the association between CTA and SPP, which are noninvasive vascular assessments used to evaluate diabetic foot wounds.

## 2. Methods

### 2.1. Study design and ethics committee approval

From November 2016 to December 2017, 52 patients with Meggitt–Wagner grade 1 diabetic foot wounds were enrolled. All of them were type 2 diabetes patients. However, 7 patients who could not undergo CTA due to renal dysfunction and 5 patients with infection at the wound site were excluded, and 40 patients were included for further analysis. In this study, the angiosome model was used to identify blood vessels affecting wounds on CTA. This study was approved by the Institutional Review Board (VHS Medical Center 2018-02-019-003).

### 2.2. The angiosome model

**Figure 1.**
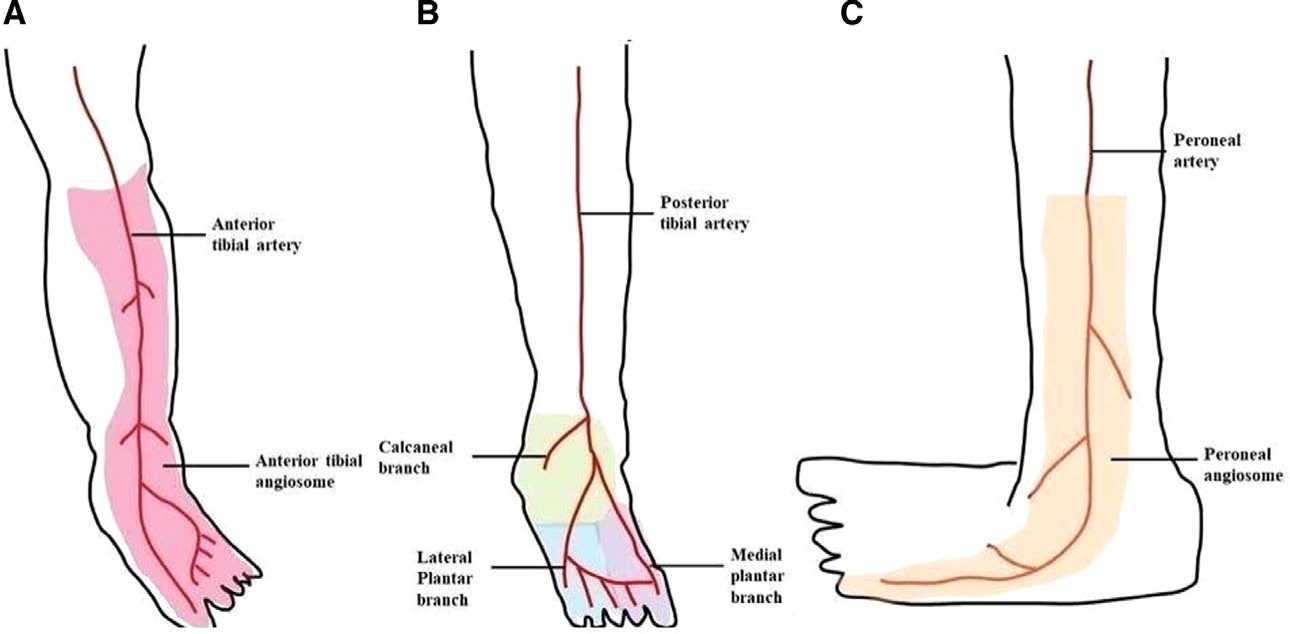
Three main arteries and 6 angiosomes of the lower leg. (A) Anterior tibial angiosome; anterior shin and dorsum of the foot. (B) Posterior tibial angiosome; medial heel and the medial and lateral plantar surfaces. (C) Peroneal angiosome; lateral aspect of the heel and the lateral border of the foot.

The angiosome represents blood vessels corresponding to specific skin regions. The lower extremities below the knee were divided into 6 zones by 3 major arteries. The posterior tibial artery supplies the medial surface of the heel and the medial and lateral plantar surfaces of the foot. The anterior tibial artery supplies the dorsum of the ankle and foot and anterior shin. The peroneal artery supplies the lateral aspect of the heel and lateral border of the foot and ankle (Fig. 1).^[9]^

### 2.3. Measurement

**Figure 2.**
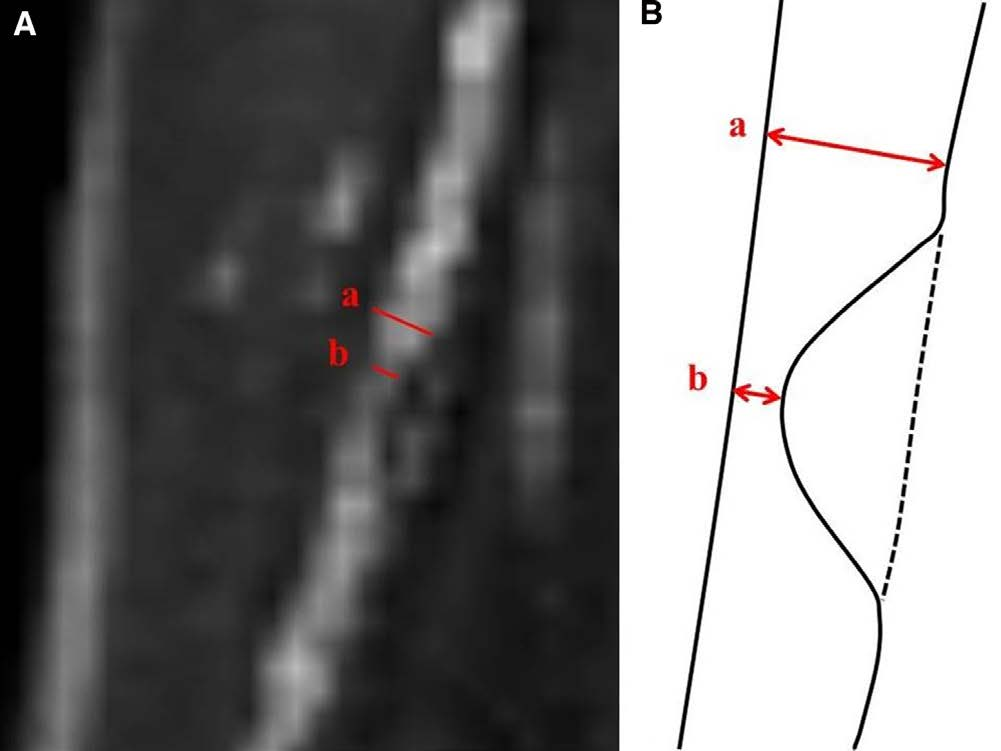
A method for measuring the degree of stenosis of a peripheral artery using NASCET is shown. (A) Measurement of the degree of stenosis in the peripheral artery corresponding to the angiosome of the wounded area in the CTA evaluation. (B) Schematic illustration of how to measure the degree of CTA stenosis. NASECT = (*a* – *b*)/*a* × 100 (where *a* is the diameter of the normal lumen; *b* is the residual lumen in a vessel with stenosis). CTA = computed tomography angiography; NASCET = North American Symptomatic Carotid Endarterectomy Trial.

The CT scanner (conventional resolution computed tomography) used in this study was Brilliance 64 (Philips Medical System); Collimation, 64 × 0.625 mm; rotation time, 420 msec; tube voltage, 100 kV or 120 kV; and tube current, 800 mA. CTA stenosis was measured by a physician working at a diabetic foot center using PACS (Picture Archiving and Communication System, INFINITT Healthcare), and the degree of vascular stenosis was classified into 4 types in the 3 blood vessels of the lower extremities: anterior tibial, peroneal, and posterior tibial arteries. The 4 types were classified as mild (0%–49%), moderate (50%–69%), severe (70%–99%), and 100% according to the North American Symptomatic Carotid Endarterectomy Trial (NASCET) criteria.^[10]^ The NASCET criteria compare the diameter minus the residual lumen from the normal lumen in the stenosis with the normal lumen diameter (Fig. 2).^[10]^

**Figure 3.**
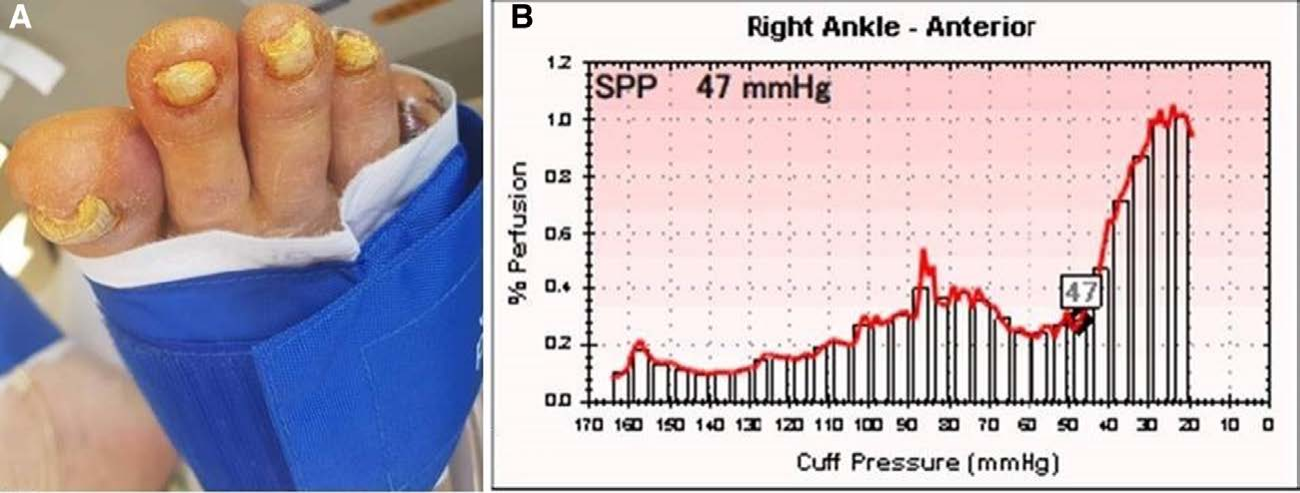
Measurement and result of SPP. (A) Measurement of the SPP in the proximal area of the wound in a patient with diabetic foot wound on the fifth toe. (B) The picture shows the result after the SPP measurement. SPP = skin perfusion pressure.

**Figure 4.**
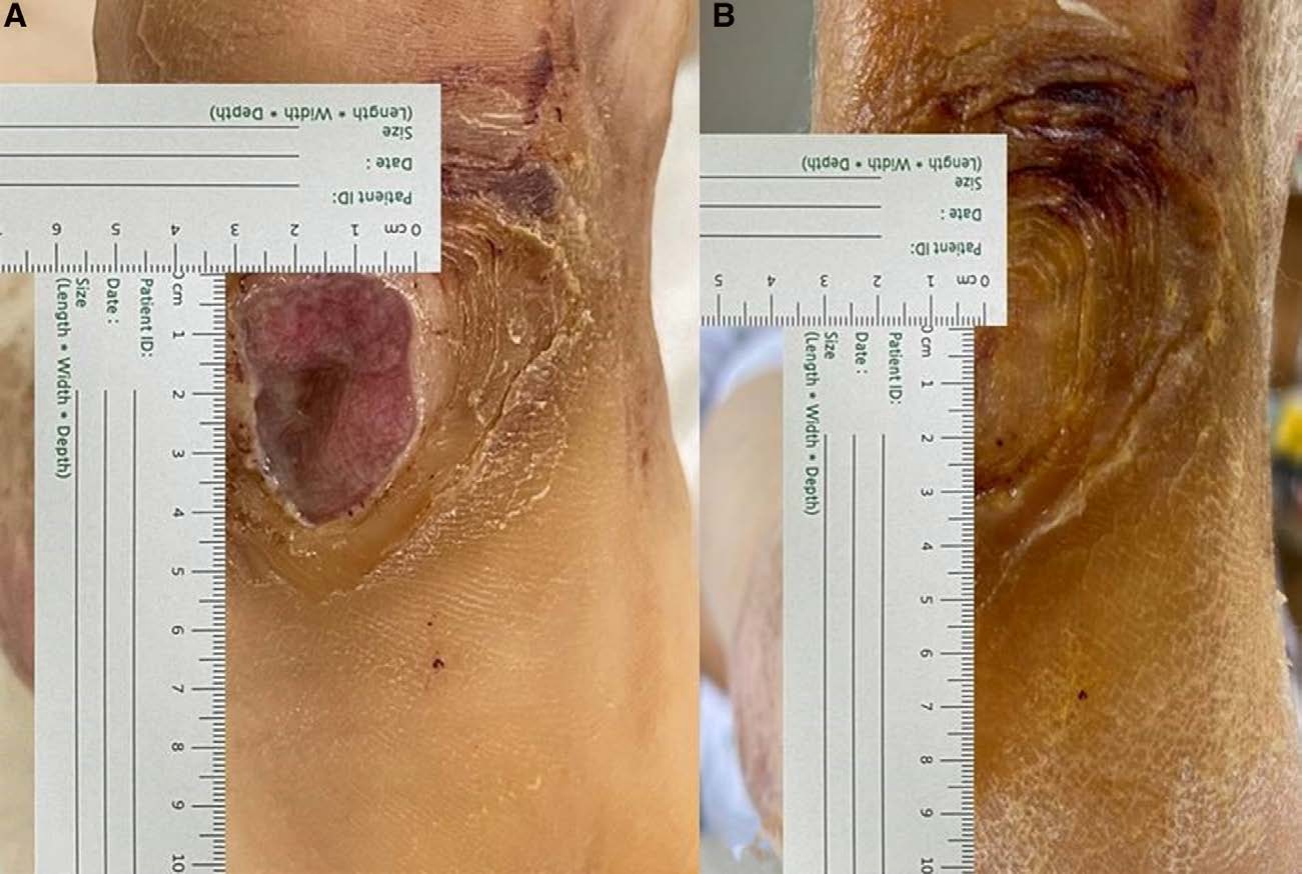
Measurement of the size of a wound. (A) Measurement of the size of a diabetic foot wound at initial treatment. (B) Measurement of the size of the diabetic foot wound after 4 wk.

The SPP used in this study was SensiLase PAD-IQ (VASAmed, USA). The SPP was measured with a laser Doppler probe beneath a blood pressure cuff placed just proximal to the wound (Fig. 3). An SPP ≥50 mm Hg was considered normal. SPPs of 30 to 50 mm Hg were considered moderate. SPP <30 mm Hg indicated a severe blood flow disorder. For wounds, 2-dimensional measurements using a ruler for wounds corresponding to grade 1 were measured at 4-week intervals (Fig. 4). If the part with the skin defect was epithelialized, it was judged to be healed, and the rate of reduction in wound size was evaluated.

### 2.4. Statistical analysis

Continuous variables are represented as mean ± standard deviation, and categorical variables are denoted as N (%). The ANOVA *P* value was used for continuous variables of CTA stenosis and SPP. Fisher exact test was used to evaluate the association between CTA stenosis and SPP. Simple linear regression was used to determine the association between the decreasing rate of wound size and the underlying disease. *P* values < .05 were considered statistically significant. Statistical analyses were performed using R (version 3.5.1; R Development Core Team, R Foundation for Statistical Computing, Vienna, Austria).

## 3. Results

Table 1 shows the averages of the measurements and demographic statistics. Of the 40 subjects, 37 (92.5%) were male and 3 (7.5%) were female. The mean age was 70.3 ± 9.0 years, 13 (32.5%) patients had hypertension, 15 (37.5%) patients were undergoing hemodialysis, and 13 (32.5%) had ischemic heart disease. The degree of CTA stenosis was mild, moderate, and severe in 22, 7, and 11 patients, respectively. SPP was normal, moderate, and severe in 7, 16, and 17 patients, respectively. Wound size was 32.4 ± 12.6 mm^2^ at the first measurement and 11.4 ± 6.4 mm^2^ at the time of measurement 4 weeks later, with a rate of reduction in wound size of 66.0%. The *P* value of the association between the degree of CTA stenosis and the SPP value was 0.915, and the *P* value of the association with the decreasing rate of wound size was 0.235. A more severe CTA stenosis indicated a lower rate of decrease in wound size. However, there was no statistically significant association between SPP and the decreasing rate of wound size according to the degree of CTA stenosis (Table 2). The association between SPP and the decreasing rate of wound size was statistically significant (*P* < .05; Table 3).

**Table 1:** Demographic data of 40 patients.

	Total (N = 40)
Age (mean ± SD)	70.3 ± 9 (range, 44.0–86.0 yr)
Sex (%) males: females	37 (92.5%): 3 (7.5%)
Underlying disease	
Hypertension	13 (32.5%)
Hemodialysis	15 (37.5%)
IHD	13 (32.5%)
CTA stenosis	
Mild	22 (55.0%)
Moderate	7 (17.5%)
Severe	11 (27.5%)
SPP (mm Hg)	
Normal	7 (17.5%)
Moderate	16 (40.0%)
Severe	17 (42.5%)
Wound size (mm^2^)	
First size	32.4 ± 12.6
Second size	11.4 ± 6.4
Decreasing rate (%/4 wk)	66.0% ± 14.4%

CTA stenosis: mild, 0%–49% obstruction of the vessel lumen; moderate, 50%–69% obstruction of the vessel lumen; severe, 70%–99% obstruction of the vessel lumen.

SPP: normal, ≥ 50 mm Hg; moderate, 30–50 mm Hg; severe, ≤ 30 mm Hg.

CTA = computed tomography angiography, IHD = ischemic heart disease, SD = standard deviation, SPP = skin perfusion pressure.

**Table 2:** Association between SPP and the decreasing rate in wound size according to the degree of CTA stenosis.

CTA stenosis				
	Mild (N = 22)	Moderate (N = 7)	Severe (N = 11)	*P* value
SPP (mm Hg)				.915
Normal	4 (18.1%)	1 (14.2%)	2 (18.1%)	
Moderate	10 (45.4%)	3 (42.8%)	3 (27.2%)	
Severe	8 (36.3%)	3 (42.8%)	6 (54.5%)	
Wound size (mm^2^)				
First wound size	32.6 ± 11.6	29.2 ± 10.9	33.9 ± 16.1	.756
Second wound size	11.2 ± 7.0	9.8 ± 5.8	12.7 ± 5.9	.659
Decreasing rate in wound size (%)	68.7 ± 14.8	67.5 ± 14.4	59.7 ± 12.7	.235

The ANOVA *P* value was used for the association between CTA stenosis and wound size, and Fisher exact test was used to determine the association between CTA stenosis and SPP.

ANOVA = analysis of variance, CTA = computed tomography angiography, SPP = skin perfusion pressure.

**Table 3:** Association between SPP and the decreasing rate in wound size

SPP				
	Normal (N = 7)	Moderate (N = 16)	Severe (N = 17)	*P* value
Wound size (mm^2^)				
First wound size	37.7 ± 18.6	31.1 ± 12.2	31.3 ± 10.3	.486
Second wound size	8.00 ± 7.7	9.75 ± 4.6	14.41 ± 6.46	.031
Decreasing rate in wound size (%)	82.2 ± 11.5	69.5 ± 6.4	56.0 ± 13.6	.000

ANOVA *P* value was used for the association between SPP and wound size.

ANOVA = analysis of variance, SPP = skin perfusion pressure.

## 4. Discussion

This study showed that SPP is more valuable than CTA as a noninvasive method to evaluate arterial status to predict the decreasing rate of wound size when noninfected foot wounds occur in diabetic patients. Tetsuya et al reported that SPP is more reliable than ankle blood pressure, toe blood pressure, and transcutaneous oxygen pressure (TcPO_2_). When these are measured together, SPP is most helpful in predicting wound healing, with an SPP of greater than or equal to 40 mm Hg with a sensitivity, specificity, and accuracy of 61.1%, 79.5%, and 74.2%, respectively.^[7]^ Takkin et al reported that SPP had a significant relationship with wound healing; however, TcPO_2_ did not show a significant relationship. SPP was also more sensitive in predicting wound healing than TcPO_2_ (89.7% vs 65.5%).^[8]^

SPP was measured using a laser Doppler sensor embedded in an inflatable pressure cuff. The cuff was placed in the proximal area around the wound, and the area where the deep structures were exposed was avoided. SPP measures the pressure at which blood flow first returns to capillaries during the controlled release of occlusive pressure.^[8]^ Watanabe et al^[11]^ reported that SPP > 30 mm Hg was required for wound healing. In contrast, Yamada et al reported that the wound healing rates were 10%, 69%, and 100% when the SPP was <40, ≥40, and ≥ 50 mm Hg, respectively. Various attempts have been made to determine the cutoff value of SPP for predicting the healing ability of ischemic wounds.^[7]^ While some reports have suggested the cutoff SPP value as 30 mm Hg,^[12]^ others have suggested a cutoff value of 40 mm Hg.^[13]^

CTA allows a rapid, high-resolution determination of arterial tree after injection of the contrast material. CTA offers the ability to view the lumen of large vessels but is limited when evaluating small calcified vessels.^[6]^ There is an ultrasonography grade for the degree of peripheral arterial stenosis in the lower extremities; however, CTA assessment of lower extremity arterial stenosis is not yet available.^[14]^ Therefore, this study applied the NASCET criteria to evaluate the degree of CTA stenosis. NASCET criteria are calculated to compare the diameter minus the residual lumen from the normal lumen in the stenosis with the normal lumen diameter in internal carotid artery stenosis.^[14]^

The lower extremities below the knee were divided into 6 zones based on 3 major arteries. Anatomical arterial flow affects wound healing. The angiosome approach ensures that arterial supply is affected by a region of ischemia.^[9]^ This study evaluated the stenosis of blood vessels in the wounded angiosome site. In the case of 2 or more sites, vessels with severe stenosis were selected.

This study used the linear technique, a simple and easy technique for measuring wound size. A method was used to measure the maximum length and the maximum horizontal width of a wound using a disposable paper ruler.^[15]^ Thomas and Wysocki.^[16]^ reported that linear measurements tend to overestimate the wound size by >40%. However, their study was deemed useful because it used the decreasing rate of wound size according to the size of the wound 4 weeks after the initial wound as data.

There was no significant association between age, sex, hypertension, hemodialysis, ischemic heart disease, and the rate of decrease in wound size (Table 4). The causes of diabetic foot wounds include repetitive trauma due to foot deformity with motor and sensory neuropathy, dry skin due to autonomic neuropathy, and peripheral arterial disease that affects wound healing.^[17]^ Glycemic control affects the improvement of neuropathy, and cardiovascular disease including hypertension, hemodialysis, and ischemic heart disease affects the progression of peripheral arterial disease.^[18]^ Therefore, the multiisciplinary approach is recommended for the treatment of diabetic foot wounds. This study focused on the effect of peripheral arterial disease on diabetic foot wounds. Therefore, it is difficult to generalize the results of this study on the treatment of diabetic foot wounds, but it is expected to be helpful in deciding how many references should be made to the selection and results of the CTA.

**Table 4:** Association between age, sex, the underlying disease, and the decreasing rate in wound size.

	*P* value
Age	.584
Sex	.433
Underlying disease	
Hypertension	.274
Hemodialysis	.118
IHD	.109

Simple linear regression was used to determine the association between the decreasing rate of wound size and the underlying disease.

IHD = ischemic heart disease.

This study was performed on Meggitt–Wagner grade 1 diabetic foot wounds, and not all patients were judged that angioplasty was necessary. Therefore, angioplasty was requested for 1 of 7 patients with moderate stenosis and 6 of 11 patients with severe stenosis in CTA with slow wound healing after 1 month of wound healing. As a limitation of this study, there may be selective errors in patients who can be followed up for 4 weeks and can be tested. Smoking, antihypertensives, blood pressure, and statins, which may have some effect on blood flow, were not investigated. Reliability may be low because there is no interobserver correlation, and the number of subjects is small.

## 5. Conclusions

The degree of stenosis on CTA, which is most commonly used for vascular status evaluation in diabetic foot wounds, had a low association with the decreasing rate of wound size but showed a significant association with SPP.

## Author contributions

Conceptualization: Euidong Yeo

Data curation: Hong Seop Lee, Yeong Yoon Koh

Formal analysis: Young Bin Shin

Supervision: Euidong Yeo

Writing—Original Draft: Hak Jun Kim

Writing—Review & Editing: Woo Jong Kim, Euidong Yeo
